# Therapeutic Approaches for ADHD by Developmental Stage and Clinical Presentation

**DOI:** 10.3390/ijerph191912880

**Published:** 2022-10-08

**Authors:** Alma Y. Galvez-Contreras, Ivette Vargas-de la Cruz, Beatriz Beltran-Navarro, Rocio E. Gonzalez-Castaneda, Oscar Gonzalez-Perez

**Affiliations:** 1 Unidad de Atención en Neurociencias, Departamento de Neurociencias, Centro Universitario de Ciencias de la Salud, Universidad de Guadalajara, Guadalajara 44340, Mexico; 2 Instituto de Neurociencias Traslacionales, Departamento de Neurociencias, Centro Universitario de Ciencias de la Salud, Universidad de Guadalajara, Guadalajara 44340, Mexico; 3 Laboratorio de Neurociencias, Facultad de Psicología, Universidad de Colima, Colima 28040, Mexico

**Keywords:** neurodevelopmental disorders, attention, hyperactivity, impulsiveness, DSM-5, neuropsychology

## Abstract

Attention Deficit Hyperactivity Disorder is a neurodevelopmental disorder with three presentations: inattentive, hyperactive/impulsive and combined. These may represent an independent disease entity. Therefore, the therapeutic approach must be focused on their neurobiological, psychological and social characteristics. To date, there is no comprehensive analysis of the efficacy of different treatments for each presentation of ADHD and each stage of development. This is as narrative overview of scientific papers that summarize the most recent findings and identify the most effective pharmacological and psychosocial treatments by ADHD presentation and age range. Evidence suggests that methylphenidate is the safest and most effective drug for the clinical management of children, adolescents and adults. Atomoxetine is effective in preschoolers and maintains similar efficacy to methylphenidate in adults, whereas guanfacine has proven to be an effective monotherapy for adults and is a worthy adjuvant for the management of cognitive symptoms. The psychosocial treatments with the best results in preschoolers are behavioral interventions that include training of primary caregivers. In adolescents, the combination of cognitive and cognitive-behavioral therapies has shown the best results, whereas cognitive-behavioral interventions are the most effective in adults. Pharmacological and psychosocial treatments must be adjusted to the ADHD presentation and its neurocognitive characteristics through the patient’s development.

## 1. Introduction

Attention-deficit/hyperactivity disorder (ADHD) is a neurodevelopmental disorder characterized by persistent inattention, hyperactivity and impulsivity behaviors [1]. ADHD is commonly diagnosed in childhood and, given its high incidence and cost of treatment, represents a public health problem throughout the world [2]. ADHD etiology is unknown, but it has a strong hereditary component (~81%) [3] and, unfortunately, 60% of the cases diagnosed during childhood will prevail into adulthood.

The Diagnostic and Statistical Manual of Mental Disorders (DSM-5) distinguishes three clinical presentations of ADHD: predominantly inattentive, predominantly hyperactive/impulsive and combined [1].

The inattentive ADHD presentation is the most commonly diagnosed (53.7%), followed by the combined type (26.8%), while the least diagnosed is the hyperactive/impulsive (19.5%) [4] (see Table 1).

Sex and age of patients are associated with ADHD presentation. In women, inattentive presentation is more frequent, while in men the combined type predominates. In contrast, the hyperactive/impulsive presentation shows similar incidences between men and women [4]. According to the stage of development, the most commonly diagnosed presentation in preschool children is the hyperactive-impulsive (2.8%) [5], followed by the combined (2.1%) and the inattentive (0.1%) [6]. In adults, the inattentive type is the most common clinical presentation of ADHD, regardless of gender [7].

The diagnosis of ADHD presentations may vary throughout the life of the patient [5]. Preschool children who meet the diagnostic criteria for hyperactive-impulsive ADHD may progress to combined-type ADHD during their elementary school, whereas children with combined-type symptoms in elementary school may shift to inattentive ADHD and their hyperactivity-impulsivity symptoms progressively decrease [5] To date, there is not enough evidence to establish whether these variations are related to the type of treatment, but it is clear that some ADHD presentations tend to be more stable than others. Lahey et al. found that children who met diagnostic criteria for the combined type maintained this presentation in subsequent evaluations, unlike children diagnosed with the hyperactive-impulsive and inattentive types, who manifested diagnostic criteria for a different presentation on at least two occasions throughout their life [8]. This variability in the diagnosis of ADHD presentation has been associated with hormonal and brain changes that occur throughout body development [3].

The first clinical manifestations of ADHD can appear from the third year of life [6]. However, the diagnosis is usually established between six and nine years of age [9]. Regardless of the ADHD presentation, the most effective treatment is multimodal, a combination of pharmacological and psychosocial approaches. Drug treatment is more effective for managing the core symptoms of ADHD (inattention, hyperactivity and impulsivity), while the psychosocial approach is more effective for treating comorbid symptoms [10]. Unfortunately, 42.2% of children diagnosed with ADHD receive a single treatment that is usually abandoned when parents or caregivers do not perceive the expected results to counteract all the academic, social and family problems associated with ADHD [11]. Therefore, it is crucial to know the most effective therapeutic approaches for each presentation and promote better adherence to treat ADHD patients. The aim of this review is to summarize the current findings of effective treatments for handling ADHD, which can help professionals to establish their clinical management. To this end, we revised the most effective pharmacological and psychosocial treatments used for ADHD in accordance with the predominant symptoms, age of patients and comorbidity. This evidence indicates that the choice of treatment should be based on the ADHD presentation, age, gender and comorbid disorder, which helps improve therapeutic adherence in these patients.

## 2. Materials and Methods

The present systematic review examines the efficacy reported in pharmacological, neuropsychological, or psychosocial treatments in ADHD according to age or presentation. We searched for articles from the National Library of Medicine by using the PubMed search engine and the terms: efficacy of neuropsychological treatments, OR pharmacological treatments, OR novel psychological treatments OR psychosocial treatments combined with the terms ADHD, ADHD-presentations, OR inattention, OR impulsivity, OR hyperactivity. 

**Figure 1:**
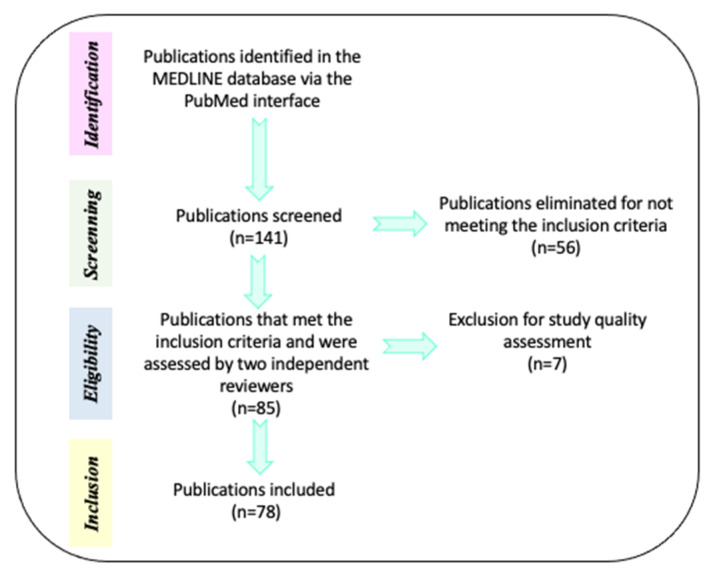
Methodological process for references selection. Description of the steps performed for the search and selection of references used for this review.

The initial search in the MEDLINE database comprised 141 papers. Of these, 56 references were eliminated because: a) they compared alternative or unregulated therapies (homeopathy, transcranial direct current stimulation, omega 3/6, fatty acids, phosphatidylserine, neurofeedback, or exercise) with pharmacological interventions and b) the efficacy of treatments (pharmacological, neuropsychological, or psychosocial) was evaluated when AHDH was comorbid with diverse neurodevelopmental disorders. Only 85 references were selected because they met inclusion criteria: a) Type of paper (clinical trials, meta-analysis, reviews, systematic reviews, randomized controlled trial, case reports, committee and government reports) and b) Efficacy of pharmacological treatments (methylphenidate—MPH-, Atomoxetine—ATM- and Guanfacine) combined or not with neuropsychological treatments (cognitive training) or psychosocial treatments (behavioral management, stress management, organization training, parental training, social skills training, cognitive therapy and metacognitive therapy) in preschoolers, children, adolescents and adults with a diagnosis of ADHD. For description of the mechanism of action for drug medication in ADHD, we also included animal models. The current review included English- and Spanish-language reports published between the years 1991 and 2021. Then, the pre-selected papers (85 documents) were subjected to methodological quality assessment by two independent reviewers. After this process, 7 reports were eliminated because they contained confirmative information, or their evidence did not approach the aim of the review. Finally, only 78 references were utilized for this review and all the selections followed PRISMA guidelines (Figure 1 and Table 2).

## 3. Results

### 3.1. Efficacy of Pharmacological Treatment According to Patients’ Development

Of the 79 selected references, 34 were used for analysis of the current pharmacological treatments. The conventional treatment for ADHD is based on the use of nervous system stimulants (amphetamines and methylphenidate) [12] and non-stimulants such as guanfacine, atomoxetine, or clonidine [13]. ADHD is the only nondegenerative mental condition where stimulant drugs alleviate the core symptoms [14]. A meta-analysis of double-blind, randomized and controlled trials reported that the combination of amphetamine and methylphenidate produces a good response in 41% of ADHD patients [15]. Nevertheless, 28% of patients show clinical improvements with the monotherapy of amphetamines and 16% with methylphenidate [15]. In children and adolescents, MPH has a higher size effect (measured through risk ratio, RR = 1.14) when compared to other drugs [16], whereas in adults the size of the effect is moderate (Standardized Mean Difference—SMD—0.50) [17]. Despite this evidence, MPH is still the first-line drug of use [18], because it has fewer adverse effects [19] and prolonged therapeutic effect (up to 24 months) after drug withdrawal [20]. However, the average use for stimulant-drug medication is low, 136 days for children and 230 days for adults, and its discontinuation is associated with a stigma about the use of medication, perception of low efficacy, multiple side effects and others [15].

After MPH oral administration, MPH reaches sufficient plasma levels in one hour [21] and blocks the dopamine and norepinephrine reuptake transporters [22] and, in turn, increases dopamine and norepinephrine synaptic levels [23], modifies dendrite length and complexity [24], promotes catecholamine-associated neural stimulation and inhibition [21] and improves neural connectivity between frontal lobe and striatum [25,26]

The long-term therapeutic effect of MPH depends on the dose, route of administration and treatment duration [27]. Seven- to eleven-year-old children diagnosed with ADHD and treated with MPH can show a significant reduction in impulsivity and clinical symptoms from the 6th to 8th week of treatment [27]. Despite that MPH is considered as a safe drug, high doses (270 mg/kg) have been associated with neurotoxicity, suicidal attempts [28,29] and paradoxical effects, such as agitation, hyperactivity and poor cognitive performance [30]. Preschool-age children (<6 years) seem to be more susceptible than older children to develop side effects, such as repetitive behaviors, recurrent thoughts, weight loss, insomnia and appetite loss [21]. Sadness and social withdrawal are additional symptoms that have also been reported in preschool-age children and are more common in the combined ADHD than inattentive presentation [6]. According to the patient’s age, in children and adolescents, MPH had the lowest rate of discontinuation in comparison to amphetamines or placebo [15]. This suggests that the ADHD presentation and the development stage of each patient seem to influence the incidence of side effects after MPH therapy. Thus, some authors recommend gradual increases in MPH doses, starting with 0.15 mg/kg followed by 0.3 to 0.60 mg/kg in children and starting with 40 mg followed by 50 to 80 mg for adults [31].

In most cases, MPH recovers cognitive performance in patients with ADHD, regardless of whether they have comorbid psychiatric disorders or not [30]. In healthy subjects, MPH improves memory performance and induces greater interest in and motivation to perform activities such as mathematics [32]. This effect of MPH on math skills has also been reported in ADHD patients (range 4 to 16 years), which is associated with clinical progression in a dose-independent matter [33], suggesting that MPH efficiently improves motivation. The pharmaceutical presentation of MPH appears to have a considerable influence on biological effects and cognitive performance. In children and adolescents, the osmotic release of MPH improves verbal fluency, selective attention, inhibitory control, spatial intelligence and working memory [27]. The long-lasting release of MPH is very effective for improving academic performance as compared to a short-term-release drug [33]. Therefore, long-lasting presentations are recommended for the management of inattentive ADHD, which is the presentation most frequently associated with academic problems.

MPH is effective for the management of oppositional defiant disorder (ODD) and reduces the incidence and severity of assaults [34]. ODD is a typical comorbid disorder of the combined ADHD presentation. Therefore, MPH is an elective drug for handling this presentation [35]. However, 25% to 35% of preschool infants (3 to 6 years old) and 25% to 78% of adults do not show clinical improvement after using MPH [19]. This evidence suggests that MPH could be more effective for the treatment of school-age children (6 to 13 years) and adolescents (14–19 years). Therefore, other non-stimulant pharmacological alternatives, such as atomoxetine or guanfacine can be recommended for the treatment of preschool and adult patients.

ATM is a norepinephrine reuptake inhibitor that increases neurotransmitter levels in the synaptic space [36]. In preschool children, ATM starting dose is 40 mg/kg and it can be increased to 80–100 mg/kg distributed over 24 h [37]. In adults, the recommended dose of ATM is 80 mg/kg, because lower doses (<60 mg/kg) have shown unfavorable therapeutic adherence and poor symptomatic control [37]. From the second week of treatment, ATM helps significantly reduce inattention, hyperactivity and impulsivity scores, but its clinical effect is more evident between the fourth and sixth week of treatment [37].

ATM is highly recommended for controlling the hyperactive/impulsive presentation in pre-escolar patients. Pharmacological presentation seems to be a significant factor that modifies the efficacy of every drug, for instance, ATM shows a similar size effect when compared to immediate-release vehicles of MPH (SMD −0.05), but a poor size effect when compared to the osmotic-release vehicle of MPH (SMD 0.031) [38]. However, some of these children may present emotional lability with this drug [6]. A physical deficit has also been reported with ATM treatment; in children (6 to 7 years) and a two-year treatment with ATM was associated with a 2.7 cm reduction in the height of the patients [15]. Therefore, the clinicians must be alert and initiate an early intervention when necessary.

Guanfacine was initially used against hypertension, but recent studies demonstrated its efficacy as an adjuvant drug for ADHD management in children and adolescents. The ideal dose of guanfacine to control ADHD symptoms is between 4 and 7 mg/kg [39]. In adults, monotherapy with guanfacine is very effective and shows a higher effect size (0.52) than placebo [40]. Guanfacine is rapidly absorbed and reaches optimal plasma concentrations in the first 4 h after oral intake. The action mechanism lies in its ability to stimulate α_2a_ adrenergic receptors in the prefrontal cortex, which facilitates neural signaling in this region [41] and reduces impulsivity [42]. Guanfacine effects are not as evident as ATM or MPH, but it significantly improves cognitive performance, attention deficit and working memory [43]. For these reasons, this drug could be prescribed as an adjuvant treatment for hyperactive/impulsive presentation, comorbid learning disabilities or ODD.

Recent studies show that the efficacy of MPH, ATM and guanfacine varies according to the stage of development of the patient. MPH has better results in the management of central symptoms in late childhood, adolescence and adulthood. ATM is less effective for the management of symptoms in late childhood and adolescence but has similar efficacy to MPH in adults [44]. Although MPH is considered an effective medication for adults, the high comorbidity of ADHD with the abuse of addictive substances in this population means that some authors recommend pharmacological treatments with non-stimulant drugs, such as ATM or guanfacine [45]. In this regard, non-stimulant drugs might be a better option for adolescents and adults with both ADHD and abuse of drugs (Table 3).

### 3.2. Efficacy of the Neuropsychological Treatment According to Patients’ Development

To summarize the findings of neuropsychological treatments, we analyzed 10 of the 79 selected references. Recently, a systematic review that compared 34 meta-analyses reported that children and adolescent patients with ADHD present a worsening in neurocognitive performance when compared with typically developing subjects [46]. Several studies have been carried out to identify the neuropsychological profile of ADHD patients and important differences have been found among presentations. These differences largely determine the type of treatment provided, which highlights the importance of understanding the neurocognitive profiles of each presentation in order to establish specific neuropsychological rehabilitation strategies for each subtype. ADHD patients showed deficits in the standardized mean difference (SMD) of cognitive functions associated with the default model network, including intelligence/achievement (SMD = 0.60), reaction time variability (SMD = 0.66), vigilance (SMD = 0.56), response inhibition (SMD = 0.52) and working memory (SMD = 0.54) [46]. In most patients, difficulties in executive functions underly the core symptoms of ADHD [47,48,49,50]. These executive impairments are associated with learning disorders and low academic achievements in 20–30% of pediatric patients [51,52]. In this regard, in children with inattentive, combined, or hyperactive-impulsive type of ADHD, the treatment based on executive-function training improves their executive skills that positively affect their routines in real daily life [53].

Dysfunction in executive functions varies for each ADHD presentation. Children and adolescents with combined or inattentive subtypes show severe deficits in the control of inhibitory responses, whereas those with the hyperactive-impulsive subtype show less executive impairment [5]. Patients with inattentive presentation also show inappropriate working memory that enhances problems in intrinsic motivation, which means that these patients get bored easily in their day-to-day tasks [50]. Hence, this evidence suggests that patients with inattentive and combined types require neuropsychological strategies of rehabilitation that promote executive skills training in behavioral inhibition, set-shifting, verbal fluency, working memory, cognitive flexibility and planning [54]. However, a meta-analysis of cognitive training reported that these treatments have a greater impact when used as an adjunctive therapy that improves some neuropsychological impairments, such as deficits in visual and verbal working memory, but they do not improve core symptoms such as inattention [55].

Other cognitive variables also present differences among ADHD presentations. Inattentive and hyperactive-impulsive types are inversely correlated to intelligence, alertness, response variability, processing speed and delay in the aversive stimulus so it is important to incorporate the evaluation and treatment of these cognitive tasks in both presentations. Notably, the predominance of inattention symptoms seems to be strongly associated with cognitive deterioration [5]. Consequently, neuropsychological treatment should be focused on correcting these symptoms and with special emphasis on women, who have the highest incidence of inattentive presentation [4] (see Table 4).

### 3.3. Efficacy of Psychosocial Treatment According to Patients’ Development

To evaluate the efficacy of psychosocial treatment in ADHD, we analyzed 21 of the 79 selected references. In this regard, the efficacy of psychosocial treatment in ADHD has been a discussion subject in the scientific literature [56]. Methodological issues such as diversity in inclusion and exclusion criteria, number of cases, or evaluation approaches can modify results and meta-analyzes, making it difficult to determine the efficacy of psychosocial treatment [57]. Despite that conflicting or contradictory results have been obtained, several psychosocial strategies have shown certain effectiveness [57,58,59,60]. Psychosocial treatments consist of cognitive intervention, social skill training, problem-solving, emotion management, academic skills [61,62] and behavioral intervention in parents and patients [63], aimed at reducing the presence of disruptive behaviors and preventing them. These treatments are mainly based on:

- Behavioral techniques, based on rewards or punishments, that seek to generate more adaptive behaviors.
- Cognitive techniques that identify maladaptive beliefs and replace them with others that generate better adaptation, i.e., cognitive restructuring or rehabilitation.
- Combination of cognitive and behavioral techniques at a group or individual level.
- Training in organizational and/or social skills.

The psychosocial intervention in patients with inattentive- or combined-type ADHD that is based on training in improving organization skills regarding school materials showed a moderate improvement in inattention [58]. In early childhood, behavioral parenting training reduces the core symptoms with a large effect size (>1) [59], whereas training in behavioral skills of parents and teachers has a large effect size for the management of ADHD in adolescents with hyperactive and impulsive symptoms [56]. In parallel, training in organizational skills of children and adolescents moderately improves their academic productivity [58]. However, this training also showed a significant effect size in task planning (d = 1.05), homework completion (d = 0.85) and academic problem solutions (d = 1.30) [56].

Cognitive interventions in children and adolescents have a moderate size of effect on improving inattention and a small effect size on controlling impulsivity and hyperactivity [64]. Similar findings have been found in adults when cognitive-behavior interventions are utilized for 2 and 4 months [65]. Interestingly, better results for improving inattention are obtained when therapy in groups is accomplished, but this approach seems not to be very effective for the management of hyperactivity and impulsivity symptoms [66]. In summary, this evidence suggests that cognitive interventions help improve inattention in children and adolescents, whereas the combination of cognitive-behavioral approaches is more effective for adults.

Up to 70% of ADHD patients have comorbid psychiatric disorders. Depression and anxiety are the internalizing comorbid disorders more prevalent in patients with inattentive and combined presentations [67,68,69,70]. Interestingly, the relationship between depression and anxiety persists throughout life in the inattentive type, but not in the combined type [71]. This may be due to the incompetence feelings that inattention usually causes, which is not as evident in the combined type. Various psychosocial treatments have been used for managing depression and anxiety. Behavioral training for parents has shown partial benefit in managing depressive and anxiety symptoms in infantile patients [72]. In contrast, cognitive-behavioral therapy notably reduces symptoms of emotional disorders in adults with ADHD [66,73]. Besides, this type of training also improves self-esteem and organizational skills [66]. Recently, acceptance and commitment therapy (ACT) is emerging as a promising treatment that improves anxiety in adults [74]. Altogether, this evidence indicates that parental behavioral training is the best approach for the treatment of depression and anxiety in children, whereas cognitive-behavioral therapy in groups seems to be the best option for adults (see Table 5).

Externalizing disorders, such as oppositional-defiant disorder, are psychopathologies that occur with a higher incidence in children with the combined presentation [75,76] and are characterized by the exteriorization of disruptive behaviors and behavioral alterations, where aggressiveness, impulsivity mad oppositionist, lack of behavioral–emotional self-control are consistent [77]. Patients with combined type have extensive externalizing symptoms [67,68,70] that provoke inappropriate social functioning [78]. Training parents in behavioral management and school-material organization have showed to improve the symptomatology of oppositional-defiant disorder, but the size of the effect is modest [64]. In 6- to 12-year-old children, training in contingency management and academic interventions are more effective inducing a significant behavioral change as compared to cognitive-behavioral strategies [59]. Therefore, behavioral interventions are recommended to control externalizing symptoms of ADHD or in patients with the combined type where ODD is frequently diagnosed.

## 4. Conclusions

In the preschool stages (3–6 years), the most prevalent presentation of ADHD is the hyperactive/impulsive subtype. In childhood (6–12 years), the combined type predominates in boys, whereas the inattentive type is the most common ADHD presentation in girls. The hyperactive-impulsive ADHD commonly progresses to a combined presentation that, in turn, evolves to the inattentive type in adults (see Table 6).

Due to cognitive and behavioral comorbidities, the most complex presentations for clinical management are inattentive and combined types. ATM is the drug with better results in preschool children because it has fewer side effects as compared to MPH. ATM is also recommended for the therapeutic management of adolescents and adults with abuse of drugs. MPH is very efficient for clinical management of late childhood and adolescents with inattentive and combined presentations because it improves working memory, intelligence, academic skills and ODD comorbidity. Guanfacine is also recommended as an adjuvant treatment in childhood and adolescents to improve academic symptoms. Guanfacine can also be useful for handling adults. In parallel, neuropsychological treatments act as adjuvant therapy, whereas the most effective psychosocial treatment in early childhood is behavioral intervention that includes training for parents.

**Figure 2:**
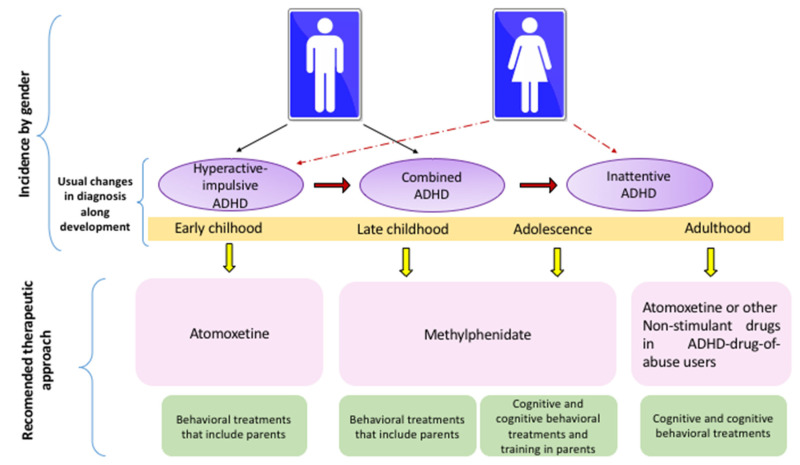
Recommended Behavioral and Pharmacological Approaches for ADHD by Developmental Stage and Clinical Presentation.

Late childhood is benefited by cognitive strategies that improve inattention, whereas cognitive-behavioral approaches reduce hyperactivity and impulsivity. In adolescents and adults, the most effective approach is the combination of cognitive and cognitive-behavioral therapies (see Figure 2).

## Figures and Tables

**Table 1:** Behavioral Expression of ADHD Subtypes.

Presentations/Subtypes of ADHD	Behavioral Expression
**Predominantly inattentive**	Do not pay attention to the details of their tasks. Make careless mistakes on homework. Have difficulty for maintaining attention to tasks or games. Seem not to hear when spoken to directly. Do not follow instructions. Do not complete schoolwork, errands or obligations. Have difficulties in organizing activities. Avoid or dislike engaging in tasks that require effort. Lose supplies or things necessary to perform activities. Easily distracted by irrelevant stimuli. Careless in daily activities.
**Predominantly** **hyperactive/impulsive**	Hyperactivity
	Move hands or feet excessively or restless when seated. Cannot remain seated when they must be seated. Run or jump excessively in situations or places where it is inappropriate. Have trouble relaxing or playing games when it is necessary to stay still. Keep permanently in motion. Excessive talking
	Impulsive
	Answer or react before questions are finished. Have difficulty queuing or waiting turns at games. Constantly interrupt conversations, games or activities.
**Combined**	Show same number of inattentive and hyperactivity/impulsivity behaviors

Typical behaviors by subtype or presentation of ADHD according to DSM-5 criteria.

**Table 2:** A summary of the main findings of each reference examined for this review.

Reference	Authors	Main Findings
1	APA	Report the diagnostic criteria for ADHD
2	National Institutes of Health Consensus Development Conference Statement	ADHD as a public health problem
3	Andersen, 2003	60% of the ADHD cases diagnosed in childhood can persist until adulthood
4	Mowlem et al., 2018	Percentage of diagnosis for each presentation in ADHD: inattention: 53.7%, combined type 26.8% and hyperactive/impulsive 19.5%
5	Willcutt et al., 2012	The most diagnosed presentation
6	Greenhill et al., 2008	The most diagnosed presentation
7	Young et al., 2013	The inattention presentation of ADHD is the most common clinical feature for adults.
8	Lahey et al., 2005	Dynamics in the diagnostic of each ADHD presentation throughout development.
9	Charach et al., 2011	Diagnosis of ADHD by age
10	Chronis et al., 2006	Importance of multimodal treatments for ADHD.
11	Hébert et al., 2013	Clinical issues that affect the therapy adherence in ADHD patients.
12	Barbaresi et al., 2007	Stimulant drugs improve reading, grade retention and school attendance
13	Caye et al., 2019	The safety of ADHD treatments should be evaluated according to individualized patients’ features.
14	Minzenberg et al., 2012	Subcellular mechanisms of pharmacological treatments in ADHD could be related to neural or cognition systems.
15	Cortese et al., 2020	The efficacy of pharmacological treatments that have been approved by regulatory agencies.
16	Liu et al., 2017	Methylphenidate shows higher response, decreases inattention and generates lower risk for adverse events as compared to Atomoxetine in children and adolescents
17	Stuhec et al., 2019	Stimulant drugs with larger size effect for ADHD treatment in adults.
18	Storebø et al., 2019	Parallel and crossover trials are suitable for analyzing the efficacy of methylphenidate in children and adolescents with ADHD.
19	Wilens et al., 2008	Role of catecholaminergic systems in the improvement of core symptoms in ADHD.
20	Huang et al., 2011	Long-term efficacy of pharmacological treatments in comparison to their adverse effects.
21	Mardomingo-Sanz et al., 2012	Effectiveness of different formulations of methylphenidate.
22	Zetterström et al., 2019	Methylphenidate efficacy depends on the duration of its administration and the brain region analyzed.
23	Pietrzak et al., 2006	Intra- or inter-individual variability may affect the methylphenidate efficacy.
24	Zehle et al., 2007	Indicates that methylphenidate could improve the core ADHD symptoms via increasing the synaptic organization.
25	Kodama et al., 2017	Methylphenidate effects on the dopamine system are brain-region dependent.
26	Schulz et al., 2017	Methylphenidate is better than atomoxetine for activating the caudate nucleus of young ADHD patients.
27	Nakanishi et al., 2017	Effects of methylphenidate and atomoxetine in the prefrontal activity of children with ADHD.
28	Ozdemir, et al., 2010	Risk of methylphenidate for developing suicide behavior.
29	Patel et al., 2017	Effects of toxic doses of psychostimulants over psychotic symptoms.
30	Cheng et al., 2014	Low doses of methylphenidate improve cognition by increasing excitatory postsynaptic currents (EPSCs), whereas high doses are related to psychosis because of EPSC blockage.
31	Huss et al., 2017	Dose optimization of methylphenidate improves the efficacy of this treatment
32	Beyer, et al., 2014	Methylphenidate as a cognitive enhancer in healthy people.
33	Kortekaas-Rijlaarsdam et al., 2017	The efficacy of methylphenidate on academic performance could be limited to math abilities.
34	Masi et al., 2017	Effectiveness of methylphenidate in aggressive behavior in ADHD plus ODD or Aggression.
35	Golubchik et al.,2019	Effectiveness of methylphenidate againts impulsive behavior in ADHD and ADHD plus ODD patients
36	Reynaud et al., 2019	Atomoxetine injections improves attentional orientation.
37	Clemow et al., 2016	Optimal doses of atomoxetine by age.
38	Rezaei et al., 2016	In children and adolescents, atomoxetine has similar size effect to methylphenidate with immediate-release vehicle, but not with the osmotic vehicles.
39	Verplaetse et al., 2019	Guanfacine as adjuvant therapy for ADHD in children and adolescents.
40	Iwanami et al., 2020	Guanfacine for the treatment of ADHD in adults.
41	Okazaki et al., 2019	Guanfacine is effective and well tolerated when compared to atomoxetine and methylphenidate.
42	Okada et al., 2019	Guanfacine reduces impulsivity.
43	Fitzpatrick et al., 2019	Guanfacine improves cognitive performance in ADHD.
44	Cortese et al., 2018	Atomoxetine is less effective for ADHD symptoms in late childhood
45	Bastiaens et al., 2019	Methylphenidate is not recommended for ADHD patients with drug addictions.
46	Pievsky et al., 2018	ADHD patients present worse neurocognitive performance as compared to neurotypical subjects.
47	Mohammadi et al., 2014	Decision-making problems in ADHD patients.
48	Barkley et al., 1997	A theoretical model which suggests that ADHD should be associated with deficits in inhibition, working memory, self-regulation and internalization of speech.
49	Castellanos et al., 2002	Executive problems in ADHD patients.
50	Diamond et al., 2005	Working memory is the main difficulty in the inattentive type of ADHD.
51	Biederman et al., 1991	ADHD in children should be categorized by comorbidity.
52	Daley et al., 2010	The core symptoms of ADHD and not the comorbid symptoms, underlaying poor academic performance.
53	Shuai et al., 2017	Training in executive functions improve daily activities in children with ADHD.
54	Bahcivan et al., 2015	Patients with inattentive-ADHD require executive skills training.
55	Cortese et al., 2015	Cognitive training improves working memory performance on children/adolescents with ADHD.
56	Chan et al., 2016	Pharmacological treatments improve the core symptoms whereas psychosocial treatments enhance academic and organizational skills in adolescents with ADHD.
57	Fabiano et al., 2015	Methodological issues found in some reports that analyze the efficacy of psychosocial treatments
58	Bikic et al., 2017	Organizational skill training improves the symptoms of ADHD In children,
59	Serrano-Troncoso et al., 2013	Psychosocial strategies show efficacy for ADHD clinical management.
60	Sibley et al., 2014	In adolescents with ADHD, behavioral therapy showed similar improvements to pharmacological approaches.
61	Burke et al., 2002	Types of psychosocial treatments.
62	Connor et al., 2002	Types of psychosocial treatments
63	Pelham, et al., 2008	Types of psychosocial treatments
64	Evans et al., 2014	Cognitive interventions show moderate size effect in children and adolescents.
65	Nakashima et al., 2021	Cognitive interventions during adulthood improve the clinical presentation of ADHD.
66	Vidal et al., 2015	Inattention symptoms improve with therapy in groups.
67	Faraone et al., 1998	Anxiety and depression are the most common comorbid problems with ADHD.
68	Mayes et al., 2009	Anxiety and depression are the most common comorbid problems with ADHD.
69	Milich et al., 2001	Anxiety and depression are the most common comorbid problems with ADHD.
70	Power et al., 2004	Anxiety and depression are the most common comorbid problems in children with ADHD.
71	Presentación & Siegenthaler, 2005	Anxiety and depression are comorbid with ADHD throughout development.
72	Van Den Hoofdakker et al., 2007	Behavioral training for parents improves the symptoms of anxiety and depression in ADHD.
73	Vidal Estrada et al., 2012	Cognitive- behavioral training improves self-esteem in ADHD patients.
74	Fullen et al., 2020	Acceptance and commitment therapy is an emerging treatment that reduces anxiety in adults with ADHD.
75	Gadow et al., 2004	Oppositional-defiant disorder is comorbid with ADHD
76	Nolan et al., 1999	Oppositional-defiant disorder are comorbid with ADHD
77	Díaz Atienza, 2006	Oppositional-defiant disorder is characterized by the exteriorization of disruptive behaviors and behavioral alterations.
78	Deault, 2010	Oppositional-defiant disorder generates social dysfunction.

Description of the main findings examined in this review.

**Table 3:** Clinical efficacy of the pharmacology therapy for controlling ADHD symptoms throughout development.

Drug	Clinical Efficacy	Developmental Stage
MPH	Inattention, impulsivity and hyperactivity.	Children and adolescents
MPH	Math skills	Children and adolescents
MPH	Motivation	Children and adolescents
MPH (Osmotic release)	Verbal fluency, selective attention, inhibitory control, spatial intelligence and working memory	Children and adolescents
MPH (long-lasting release)	Academic performance	Children
MPH	Control incidence and severity of ODD assaults	Young
ATM	Hyperactive/impulsive	Preschool child
Guanfacine	Cognitive performance, attention deficit, hyperactivity and working memory	Children, adolescents and adults

Main effects of pharmacological therapy in ADHD.

**Table 4:** Neuropsychological Treatments Evidence in ADHD.

Cognitive Training	Efficacy on Symptoms	Developmental Stage
Executive training	Executive skills and improve routines in real daily life	Children
Cognitive training	Improve deficits in visual and verbal working memory	Children

**Table 5:** Psychosocial Treatments Evidence in ADHD.

Psychosocial Managements	Externalizing Symptoms	Internalizing Symptoms	Inattention	Impulsivity	Hyperactivity	Development Stage
Behavioral therapy	−	+	−	−	−	Children
Behavioral parenting training	−	+	+	+	+	Children and adolescents
Cognitive interventions	−	−	+	−	−	Children, adolescents and adults
Cognitive/behavioral therapy	−	+	−	−	−	Adults
Cognitive therapy for groups	−	−	+	−	−	Adults
Organization training	−	−	+	−	−	Children and adolescents

Psychosocial treatments utilized in ADHD patients and their efficacy by predominant symptoms: internalizing (depression, anxiety and somatic disorders), externalizing (oppositional, antisocial, etc.) and central (inattention, hyperactivity and impulsivity). Effective (+) and not effective (−).

**Table 6:** Recommended Behavioral Therapeutic Approaches for ADHD by Developmental Stage and Clinical Presentation.

Stage of Development	ADHD Innatentive	ADHD Combined
Childhood and adolescence	Behavioral training for parents.	Behavioral training for parents.
	Training in organization of school supplies.	Training in organization of school supplies.
	Cognitive intervention.	Cognitive intervention.
	Behavioral management in classroom.	Behavioral management in classroom.
	Peer behavioral intervention.	Peer behavioral intervention.
	Organizational skills training.	Organizational skills training.
	Combination of behavioral treatments for parents and teachers.	Combination of behavioral treatments for parents and teachers.
		Strategies for contingency management and academic interventions.
Adulthood	Cognitive behavioral therapy.	Cognitive behavioral therapy.
	Cognitive training.	Cognitive training.
	Metacognitive therapy.	Metacognitive therapy.
	Cognitive behavioral therapy.	Cognitive behavioral therapy.
	Cognitive rehabilitation.	Cognitive rehabilitation.

Psychosocial treatments by developmental stage and subtype/presentation of ADHD.
